# Age-Friendly Care, PA: A Collaborative for Age- and Dementia-Friendly Care

**DOI:** 10.1093/geroni/igaa057.2591

**Published:** 2020-12-16

**Authors:** Erica Husser, Donna Fick, Judith Hupcey, Jenny Knecht Fredo

**Affiliations:** 1 Penn State, Spring Mills, Pennsylvania, United States; 2 Penn State, University Park, Pennsylvania, United States; 3 Penn State College of Nursing, University Park, Pennsylvania, United States

## Abstract

The Penn State College of Nursing (including the Center for Geriatric Nursing Excellence [CGNE] and Center for Nursing Research [CNR]) has partnered with the Primary Health Network (PHN) to work collaboratively toward implementing the 4Ms framework of an Age-Friendly Health System at PHN’s primary care sites in Pennsylvania. PHN is the largest FQHC in the state and spans 16 counties in PA and 2 in Ohio. Twenty of PHN’s PA primary care sites are in rural counties, and both rural and urban sites serve older adult populations with major health disparities. Connecting primary care practices with local community resources and programs is an important step in serving rural populations, and we have used the ECHO model to help facilitate connections. This session will focus on how we are using the ECHO model to engage our partners in a collaborative learning environment.

